# 

**Published:** 1915-03-06

**Authors:** 


### CONTENTS.

PAGE

**The Public Relations of Post-Mortem Examinations** ... ... ... ... ... 499

**The Clinical Value of Poor-Law Infirmaries**... 500

**Hospital and Institutional News** ... ... ... ... 501
Payment for Wounded Soldiers: Official Statement—Bombardments and Annual Reports—The German Hospital since the War—North Evington Infirmary: The Guardians' Decision—The Disadvantages of Schools as Temporary Hospitals—The New Medical School at Cardiff—Remarkable Hospital and Red Cross Chapels—The Nuisance at Southwark Infirmary—The Effect of Marriage on the Mortality of Women—The Outside Teaching of Midwives—A Testator on Investing Legacies—Casualties in the French Medical Service—The Liverpool Portable Hospital—The Selection of Sanatoria Cases: A New Whole-time Post—The Site of St. Katharine's—Another Scheme held up by the War—The Hospital for Indians at Brockenhurst—Women Doctors and the Wounded—Cottage Hospitals and the War—Winwick Mental Hospital for Wounded Soldiers—A Readjustment of Salary—The League of Mercy and the War—"The Institutional Worker"—This Week's Drug Market.

PAGE

**A Debate on Cerebro-Spinal Meningitis** ... 507
The Problems of Military Epidemics.

**St. George's Hospital and the Wounded** ... 508

**The Work of Radium Institutes** ... ... ... ... 509
The Cases Received and the Results of Treatment.

**The Electro-Cardiograph** ... ... ... ... ... 510
Laboratory Findings and Clinical Applications.

**Developments at the London Hospital** ... 511
Points in the Quarterly Report.

**The Evolution of the Modern Hospital** ... 512
II.—The Nightingale School and a New Epoch.

**A Medical Causerie** ... ... ... ... ... 513

**Round the World** ... ... ... ... ... 514

Fellow-Workers and the Roll of Honour.

**The Investigation of England's Vitality** ... 515
Actual Results of Public Health Work.

**The Editor's Letter-Box** ... ... ... ... 517
The Education of Hospital Officers—"The Royal College of Surgeons Ward"

**King Edward VII.'s Hospital, Cardiff** ... ... 518

**Institutional Pharmacists and the "B.P."** ... 518

[*See over.*]

### CONTENTS—(*continued*).

| | PAGE |
|---|---|
| **The Institutional Library** ... ... ... ... | 520 |
| Handbook for Orderlies—A Sanitary Inspector's Manual—Modern Hæmatology. | |
| **Medical Appointments** ... ... ... ... | 522 |
| **Personalia** ... ... ... ... ... ... | 522 |
| **Buildings Contemplated** ... ... ... ... | 522 |
| **Institutional Needs** ... ... ... ... ... | 522 |
| **The Institutional Worker** ... ... (Suppl.) | 1 |
| Institutional Workers and their Work: 1. The Hospital Secretary. | |
| Question for March. | |
| War Matters. | |
| Employment and Training. | |
| The Housekeeper's Department. | |
| Miscellaneous. | |
| Acknowledgments. | |

